# Dual-Porosity (Ta_0.2_Nb_0.2_Ti_0.2_Zr_0.2_Hf_0.2_)C High-Entropy Ceramics with High Compressive Strength and Low Thermal Conductivity Prepared by Pressureless Sintering

**DOI:** 10.3390/ma16062495

**Published:** 2023-03-21

**Authors:** Qian Yang, Cuiyan Li, Haibo Ouyang, Ruinan Gao, Tianzhan Shen, Jianfeng Huang

**Affiliations:** Key Laboratory for Green Manufacturing & Functional Application of Inorganic Materials, School of Materials Science and Engineering, Shaanxi University of Science and Technology, Xi’an 710021, China

**Keywords:** high-entropy carbides, pressureless sintering, porous ceramics, thermal conductivity, compressive strength

## Abstract

Porous (Ta_0.2_Nb_0.2_Ti_0.2_Zr_0.2_Hf_0.2_)C high-entropy ceramics (HEC) with a dual-porosity structure were fabricated by pressureless sintering using a mixture powder of ceramic precursor and SiO_2_ microspheres. The carbothermal reduction in the ceramic precursor led to the formation of pores with sizes of 0.4–3 μm, while the addition of SiO_2_ microspheres caused the appearance of pores with sizes of 20–50 μm. The porous HECs exhibit competitive thermal insulation (4.12–1.11 W·m^−1^ k^−1^) and extraordinary compressive strength (133.1–41.9 MPa), which can be tailored by the porosity of the ceramics. The excellent properties are ascribed to the high-entropy effects and dual-porosity structures. The severe lattice distortions in the HECs lead to low intrinsic thermal conductivity and high compressive strength. The dual-porosity structure is efficient at phonon scattering and inhabiting crack propagations, which can further improve the thermal insulation and mechanical properties of the porous HECs.

## 1. Introduction

Porous ultra-high temperature ceramics (UHTCs) are considered as promising materials for aerospace thermal protection systems (TPS) [1,2,3] because they not only possess the inherent property of UHTCs (such as high melting point, outstanding ablation resistance, and excellent high temperature stability) [4,5,6,7,8] but also present the distinguished characteristics of porous ceramics (such as low density and low thermal conductivity) [9,10,11].

Significant efforts have been made to tailor the thermal conductivity and compressive strength of porous UHTCs to meet the strict requirements for aerospace TPS. Various methods have been employed in the fabrication of porous UHTCs, including partial sintering [12], sol-gel [13], direct foaming [14], freeze-drying [15], and template methods [16,17]. Among these methods, the template method is attractive for fabricating porous UHTCs with controllable pore structure, tunable compressive strength, and thermal conductivity. Wang et al. [18] prepared porous ZrB_2_-SiC ceramics by a partial sintering method using KCl as the space holder. The KCl content and particle size could tune the porosity of these ceramics. The porous ZrB_2_-SiC ceramics exhibited high porosity (45–67%), low average pore sizes (3–7 μm), high compressive strength (32–106 MPa), and low thermal conductivity (13–34 W·m^−1^ k^−1^). Fu et al. [19] reported a porous ZrC ceramic prepared using carbon black as a sacrificial template. The porosity of these ceramics ranged from 12.47 to 4.83 μm and could be regulated by the sintering pressure. At the same time, the compressive strength decreased from 31.3 to 5.7 MPa, and thermal conductivity decreased from 2.8 to 0.5 W·m^−1^ k^−1^. Zhao et al. [20] prepared porous ZrC ceramics by adopting ZrC hollow microspheres (HMs) as the pore-forming agent to form submicron pores in the ceramic. They found that the pore structure of the porous ZrC ceramics is complex. Besides the pores formed by ZrC HMs, there are also many pores formed by the powder overlapping during the sintering process. The complex pore structure led the porous ZrC ceramics to achieve excellent thermal insulation at relatively low porosity.

Besides the effort in controlling pore structure, developing novel material is another way to meet the strict requirements of the TPS. Recently, high-entropy ceramics (HECs) have drawn significant attention from researchers due to their low thermal conductivity, good high temperature stability, slow diffusion rate, and serious lattice distortion [21,22,23,24]. Higher mixing entropy promotes the formation of single-phase solid solutions and leads to more homogeneous organization and higher thermophysical properties [25,26,27,28]. The emergence of high entropy ceramics (HECs) offers more possibilities to modulate the performance of ultra-high temperature materials and to overcome bottlenecks in material applications [29,30,31,32]. High-entropy carbides (HECs), such as (Ta_0.2_Nb_0.2_Ti_0.2_Zr_0.2_Hf_0.2_)C, (Ta_0.2_Nb_0.2_Ti_0.2_Zr_0.2_Hf_0.2_)B-2, (Ti, Zr, Nb, Ta, Mo)C, and (Ti, Ta, Nb, Zr, W)(C,N), are considered to be potential UHTCs for TPS because they exhibit lower thermal conductivity, higher mechanical strength, hardness, melting point, and thermal stability than single transition metal carbide components such as ZrC, HfC, NbC, TaC, and TiC [33,34,35,36,37].

In this work, porous (Ta_0.2_Nb_0.2_Ti_0.2_Zr_0.2_Hf_0.2_)C high-entropy ceramics were prepared by pressureless sintering with the in situ carbothermal reduction method. SiO_2_ microspheres were employed as pore-forming agents to tailor the porosity and pore structure. The effect of SiO_2_ content on the microstructure, comprehensive strength, and thermal conductivity of the obtained HECs was discussed. The fabrication method reported in this work is a universal approach to preparing HECs with controllable pore structure, tunable compressive strength, and thermal conductivity. The obtained HECs, possessing low thermal conductivity and superior compressive strength, can be competitive materials for TPS applications.

## 2. Materials and Methods

### 2.1. Materials

Commercially available tantalum chloride (TaCl_5_, 99.9%), niobium chloride (NbCl_5_, 99.9%), titanium chloride (TiCl_4_, 99.9%), zirconium chloride (ZrCl_4_, 99.9%), and hafnium chloride (HfCl_4_, 99.9%) were purchased from Shanghai Aladdin Biochemical Technology Co., Ltd. (Shanghai, China). Furfuryl alcohol (C_5_H_6_O_2_, 99.0%) was employed as the carbon source, and ethanol (C_2_H_6_O, analytical reagent) was used as the solvent. Silica microspheres with a diameter of 10 μm were employed as the pore-forming agent.

### 2.2. Synthetic Procedure

**Figure 1:**
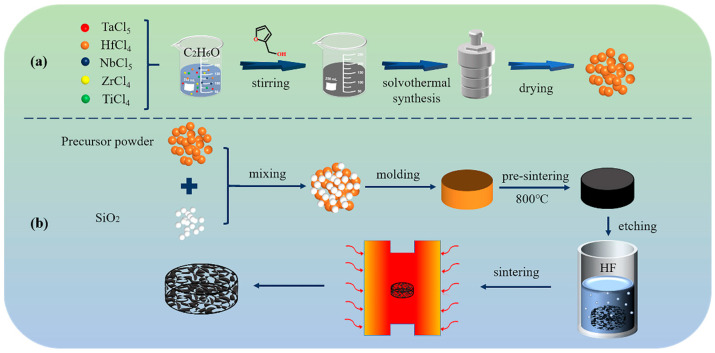
Schematic diagram of the preparation process of (**a**) precursor powders, (**b**) porous HECs.

The synthesis procedure of the precursor powder is illustrated in Figure 1a. TaCl_5_ (0.05 mol), NbCl_5_ (0.05 mol), TiCl_4_ (0.05 mol), ZrCl_4_ (0.05 mol), and HfCl_4_ (0.05 mol) were dissolved in 500 mL ethanol to obtain a transparent solution. Furfuryl alcohol (10 mL) was added to the metal chloride solution and stirred in a water bath at 60 °C for 30 min to obtain a precursor solution. Then, the precursor solution was transferred to a Teflon-lined autoclave for a solvothermal reaction at 200 °C for 12 h. Finally, the product was centrifuged and dried to obtain the precursor powder.

Figure 1b exhibits the fabrication process of the porous HECs. The precursor powder was mixed with different amounts of SiO_2_ microspheres to obtain the porous HECs with various porosity. The samples with 0, 5, 10, 15, and 20 wt.% of SiO_2_ microsphere content are called HEC-1, HEC-2, HEC-3, HEC-4, and HEC-5, respectively, in the forthcoming discussion. The mixed powder was uniaxially pressed into a cylindrical body with a diameter of 30 mm under a pressure of 15 MPa. Then, the prepared green bodies were heat-treated at 800 °C for 2 h in an argon atmosphere. The pre-sintered samples were immersed in hydrogen fluoride (HF) solution (15%) to etch the SiO_2_ microspheres. Finally, the etched samples were sintered at 2100 °C for 2 h in an argon atmosphere.

### 2.3. Characterization

The phase compositions of the samples were characterized by X-ray diffraction (XRD, D8ADVANCE-A25, Bruker, MA, USA). The microstructures of the samples were analyzed by aberration-corrected high-resolution transmission electron microscopy (HRTEM, JEM-ARM300F, JEOL, Kyoto, Japan) equipped with electron energy dispersive spectroscopy. The morphologies of the samples were observed by a scanning electron microscope (SEM, Nova Nanosem 430, FEI, OR, USA).

The thermal insulation properties of porous HECs were characterized by the thermal conductivity (*k*) values, which were calculated according to Equation (1).

(1) $$k=\alpha \cdot \rho \cdot C_{p}$$

where *α* is the thermal diffusivity, *ρ* is the density of the material, and *C_p_* is the specific heat capacity. The thermal diffusivity (*α*) was measured by a laser thermal conductivity testing instrument (LFA 467 HT, NETZSCH, BAV, German) using a sample with a diameter of 12.7 mm and a thickness of 2 mm. Specific heat capacity (*C_p_*) was measured on a differential scanning calorimeter (DSC 250, TA, NETZSCH, BAV, German). Argon was used as the test atmosphere, and the heating rate was set to 10 K/min, with the temperature range from room temperature to 1200 °C. The pore-size distributions and porosity were analyzed by mercury porosimetry (AutoPore IV 9500, Micromeritics, Norcross, GA, USA). The compressive strength of the samples was measured by a universal testing machine (CMT6103, MTS, Eden Prairie, MN, USA). The data for compressive strength represents the average volume of three specimens.

## 3. Results and Discussion

**Figure 2:**
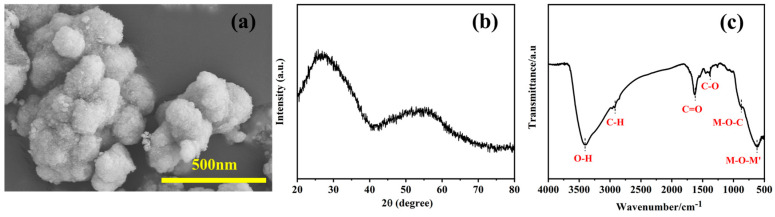
(**a**) SEM image, (**b**) XRD pattern, and (**c**) infrared spectrum of the precursor powder obtained by the solvothermal method.

Figure 2a shows the morphology of the submicron precursor particles. The XRD pattern (Figure 2b) shows the precursor was amorphous carbon structure without oxides segregation. The precursor powder was composed of nanospheres with diameters of ~200 nm. These nanospheres were made up of fine particles with a diameter of 2 nm and aggregated to form a loose structure. The fine and loose structures of the nanosphere precursor powder facilitated the plasticity and compressibility in the molding process. The chemical bonding of the precursor powder is shown in Figure 2c. O-H, C-H, C=O, and C-O bonds can be observed, indicating the polymeric state of the precursor’s powder. Furthermore, the M-O-C bonds and M-O-M’ (M’=Ta, Nb, Ti, Zr, and Hf) bonds can be found in the samples, suggesting the occurrences of the condensation reactions during the solvothermal reaction. M-O-C and M-O-M’ (M’=Ta, Nb, Ti, Zr, and Hf) bonds can promote the carbothermal reduction in the precursors in the sintering process [38].

**Figure 3:**
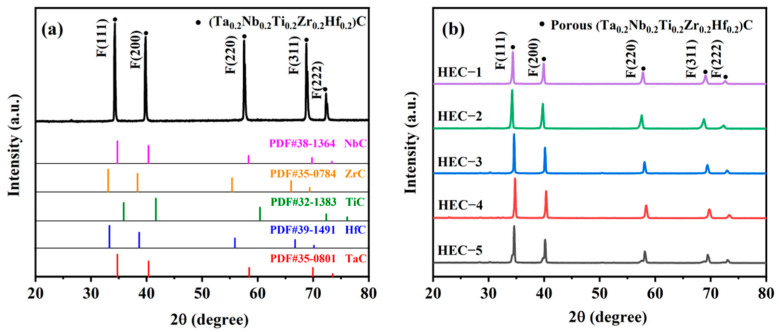
XRD patterns of obtained porous HECs: (**a**) comparison of HEC-1 sample with various carbides, (**b**) comparison of different porous HECs with various amount of pore-forming agent.

Figure 3a shows the phase structure of the HEC-1 sample. The diffraction positions of the HEC sample were referred to the X-ray diffraction patterns of TiC (PDF: 32–1383), ZrC (PDF: 35–0784), NbC (PDF: 38–1364), HfC (PDF: 39–1491), and TaC (PDF: 35–0801). The HEC exbibits a single cubic phase typical for HEC carbides with a lattice parameter of 4.51 Å in the (111) plane corresponding to those previously reported for this composition. The diffraction peaks of the HEC-1 sample are located around the corresponding diffraction peaks of the five carbides. This further suggests that the five different metal elements form the carbide solid solution. Furthermore, there is no peak for oxide in the XRD pattern. A tiny peak around 26° can be found in the XRD pattern of the HEC-1 sample, which is due to the excess carbon in the precursor powder. Figure 3b presents the XRD patterns of different HEC samples with various amounts of pore-forming agent. There is a slight difference in the diffraction positions among the other samples. The lattice plane values of the HEC samples were calculated as 4.5102 Å (HEC-1), 4.5267 Å (HEC-2), 4.4892 Å (HEC-3), 4.4681 Å (HEC-4), and 4.4827 Å (HEC-5), which are close to the HECs reported values in the reference [32].

**Figure 4:**
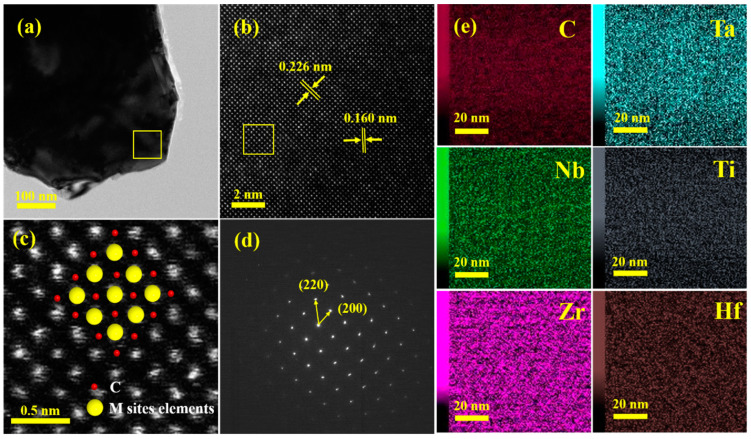
(**a**) TEM image; (**b**) HAADF image; (**c**) schematic diagram of the atom; (**d**) SAED pattern of HEC-1 sample; (**e**) EDS mappings for elements C, Ta, Nb, Ti, Zr, and Hf shown in the yellow border in (**a**).

Figure 4a,b show the transmission electron microscopy (TEM) and high-angle annular dark field (HAADF) images of the HEC-1 sample. The HAADF image shows complete and continuous periodic lattice fringes, and the interplanar spacing is measured as 0.226 and 0.160 nm, corresponding to the (200) and (220) planes of the FCC structure. As shown in Figure 4c, since the brightness in the HAADF image is approximately proportional to the square of the atomic number in the periodic table, the bright spot in the image is the location of the transition metal atom with a large atomic number. Moreover, the sample quickly absorbs the light for the C atom with a smaller atomic number, resulting in a black spot at the position of the C atom in the HAADF image. The selected area electron diffraction (SAED) pattern further elaborates the cubic crystal structure of the obtained HEC shown in Figure 4d. Figure 4e is the energy dispersive spectrometry (EDS) map of the yellow border in Figure 4a. The six elements Zr, Hf, Ti, Nb, Ta, and C are uniformly distributed in the material without evident segregation, which further verifies the successful preparation of high-entropy (Ta_0.2_Nb_0.2_Ti_0.2_Zr_0.2_Hf_0.2_)C.

**Figure 5:**
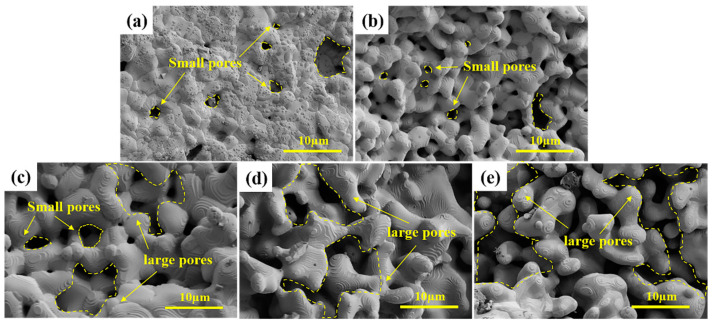
SEM images of the porous HECs with various amounts of pore-forming agents (**a**) HEC-1, (**b**) HEC-2, (**c**) HEC-3, (**d**) HEC-4, and (**e**) HEC-5.

**Figure 6:**
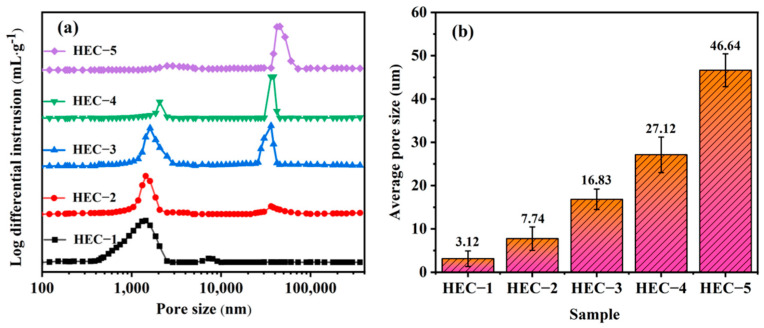
(**a**) Pore-size distributions and (**b**) average pore diameters of the porous HECs with the various amounts of pore-forming agent.

The morphologies of porous HEC samples with different amounts of pore-forming agent are displayed in Figure 5. Most of these pores in the HEC-1 sample are smaller than 2 µm. These small pores were generated from multiple particles sintering and CO gas produced in the carbothermal reaction process. With the addition of pore-forming agent, the morphology of the porous HECs changed significantly. Except for these small pores, spacing can be found among HEC grains. The size of these spacings increases with the increase in the pore-forming agent. The pore-forming agent generates these spacings, forming large pores in the porous HECs. Mercury porosimetry is used further to understand the porosity structure of the porous HEC. The porosities of porous HEC samples are listed in Table 1, and the pore-size distributions are presented in Figure 6a. The porous HECs exhibit a typical dual-porosity structure. The first type of pores is small, ranging from 0.4 to 3 µm. The second kind of pores is the large pores ranging from 20 to 50 µm. The porosity of the porous HECs grows linearly from 23.08% to 59.34% with the increase in the amount of pore-forming agent, while the average pore sizes increase from 3.12 µm to 46.64 µm. Furthermore, the small pores gradually disappeared with the rapid growth of the large pores with the increase in the amount of pore-forming agent. The mercury porosimetry results correspond to the morphology analysis. It suggests that the pore-forming agent controls the porosity volume and tailors the pore structure of the porous HECs.

**Figure 7:**
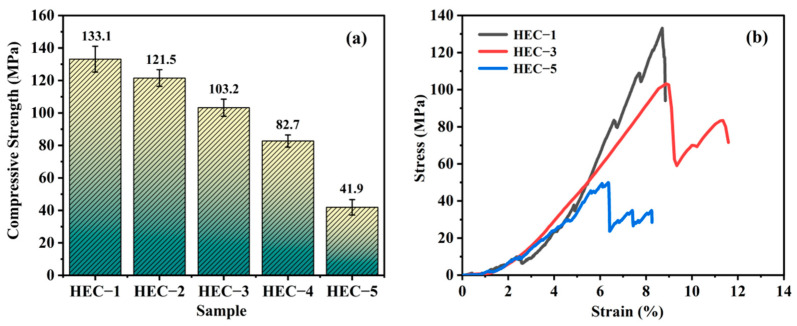
(**a**) Relationship between compressive strength and porosity, and (**b**) typical stress-strain curves of the porous HECs.

Figure 7a shows the compressive strength of the porous HECs with various porosity values. The compressive strength of the porous HECs decrease with the increase in porosity. Mainly, with the rise of porosity from 23.08% to 59.34%, the compressive strength decreases from 133.1 MPa to 41.9 MPa. Figure 7b shows the typical compressive strain-stress curves of the porous HECs. The HEC-1 sample exhibits a specific brittle fracture characterization, in which the porous ceramic ruptures when the stress surpasses the maximum value. Several zig-zag patterns can be found in the corresponding curve before the rupture, which is attributed to the squashing of the small pores. The rupture of the pores causes cracks that eventually fracture the ceramics. In contrast, the zig-zag pattern occurs after the fracture for both the HEC-3 and HEC-5 samples. The drop in the stress for both the HEC-3 and HEC-5 samples is more significant than that of the HEC-1 sample. The fractures in the HEC-3 and HEC-5 samples mainly respond to the crack propagations among the larger pores. The HEC-5 sample presents a smaller compressive stress, and strain upon fracture. However, the HEC-3 sample shows similar strain and slightly less stress than the HEC-1 sample. The excellent compressive strength is due to the dual-porosity structure of the HEC-3 sample. The small pores can elongate the crack propagation path and enhance the compressive properties.

**Figure 8:**
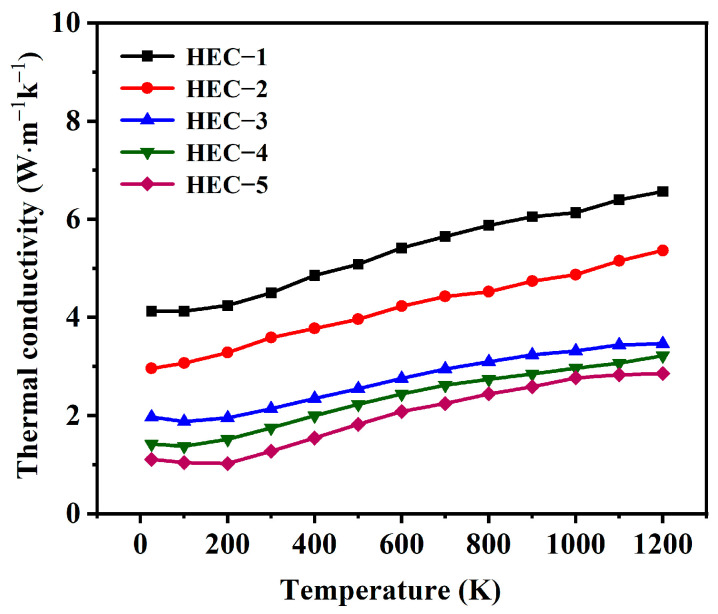
Thermal conductivity of porous HECs with different amounts of the pore-forming agent.

The thermal conductivity of porous HECs with different amounts of the pore-forming agent are shown in Figure 8. It can be seen that porous HECs exhibit low thermal conductivity in the temperature range of 25–1200 °C. The thermal conductivity increases with the increase in temperature. It is known that the heat transfer through lattice vibrations decreases, and convection heat transfer increases with the rise of temperature [20]. The increase in thermal conductivity with temperature indicates that the convection heat transfer is an important heat-transfer mechanism of porous HECs.

The thermal diffusivity (*α*), specific heat (*C_p_*), and room temperature thermal conductivity (*k*) values of these samples are shown in Table 2. The room temperature thermal conductivity ranges from 4.12 W·m^−1^ k^−1^ to 1.11 W·m^−1^ k^−1^, which is lower than dense HECs (6.45 W·m^−1^ k^−1^) due to high porosity. Comparing the thermal conductivity of HEC-1 to HEC-5 at room temperature, the thermal conductivity of porous HECs decreases rapidly when the porosity increases from 23.08% to 36.92%. However, the drop in the thermal conductivity becomes flat when the porosity exceeds 36.92%. The relationship between the thermal conductivity and porosity of the obtained porous HEC is inconsistent with the classical theoretical models of thermal conductivity, such as series [39], parallel [40], Maxwell–Eucken [41], and effective medium theory [42], because these theoretical models are based on only one kind of given porous structure. This discrepancy can also be found in the porous ZrC ceramics prepared using ZrC hollow spheres as the pore-forming agent [20]. This abnormal thermal conductivity behavior was ascribed to the complex pore structure in the porous ceramics. As discussed earlier, the porous HECs possess a dual porosity structure. Besides the large pores (20–50 µm) formed by SiO_2_ pore-forming agent, several small pores (0.4–3 µm) were also created by the grain overlapping during the sintering process. Wang et al. [41] developed a universal model that is reasonable for estimating the thermal conductivity of heterogeneous materials with multiple continuous phases. According to the universal model, the effective thermal conductivity *K_e_* of the porous HEC can be calculated by Equation (2) with suitable parameters di and k′.

(2) $$K_{e}=\frac{\sum_{i=1}^{2}k_{i}v_{i}\frac{d_{i}k^{\prime }}{\left(d_{i}-1\right)k^{\prime }+k_{i}}}{\sum_{i=1}^{2}v_{i}\frac{d_{i}k^{\prime }}{\left(d_{i}-1\right)k^{\prime }+k_{i}}}$$

where *k* is the thermal conductivity of the component, *v* is the volume fraction of the component, *d_i_* is the shape factor, and *k′* is the effective thermal conductivity of the structure. The obtained porous HECs can be considered spherical dispersed phase structures in this work. The most common approach is to take *d_i_* = 3. Since HECs are only in the solid phases for porous materials, components 1 and 2 can be regarded as dense HEC and helium, and the thermal conductivities at room temperature are 6.45 and 0.0579 W·m^−1^ k^−1^, respectively. The testing data in this work are consistent with the universal model when *k′* is chosen as 1.66. This suggests that the universal model can predict the thermal conductivity of porous HECs with a complex pore structure.

**Figure 9:**
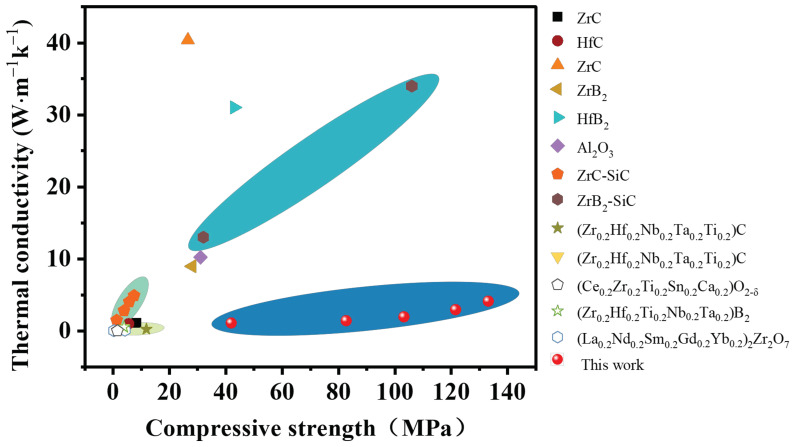
Comparison of the compressive strength and thermal conductivity of the obtained porous HECs with other porous UHTCs from previous reports [18,30,31,43,44,45,46,47,48,49,50,51].

Figure 9 illustrates the properties of compressive strength and thermal conductivity of the porous HECs in this study and UHTCs reported in the literature. Compared with these porous UHTCs, the porous HECs in this study achieved good thermal insulation and excellent compressive strength. Notably, the porous HECs are competitive in thermal insulation while possessing extraordinary compressive strength.

**Figure 10:**
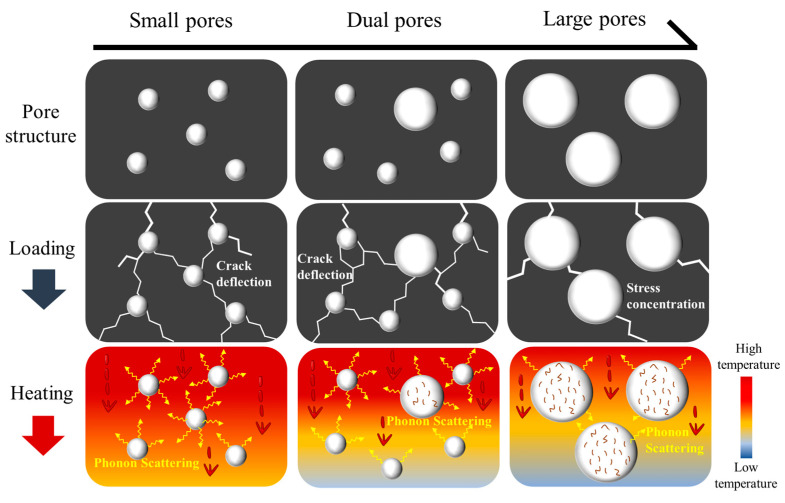
Pressure resistance and thermal conductivity mechanism diagram of dual-porosity high-entropy ceramics.

The excellent properties of the porous HECs can be explained by the schematic diagram shown in Figure 10. The good thermal insulation and excellent mechanical properties of the porous HECs come from the synergistic effect of high-entropy and dual-porosity structures. The differences in atomic radii and bond strength of these five metal cations (Ti, Zr, Ta, Hf, and Nb) lead to the atomic-level lattice distortion in the (Ta_0.2_Nb_0.2_Ti_0.2_Zr_0.2_Hf_0.2_)C ceramics. The severe lattice distortions reduce the mean free path of phonons and increase phonon scattering, resulting in low intrinsic thermal conductivity in high-entropy ceramics [32,52]. The room temperature thermal conductivity of the densified (Ta_0.2_Nb_0.2_Ti_0.2_Zr_0.2_Hf_0.2_)C high-entropy ceramics is only 6.45 W·m^−1^ k^−1^, which is much lower than that of the solid ZrC [32]. Moreover, the severe lattice distortion blocks the crack propagation, resulting in a significant increase in compressive strength [53].

The number of pores in the ceramics increases the probability of phonon collisions, decreasing the mean free path of gas molecules. Eventually, it reduces the phonon thermal conductivity. Therefore, the thermal conductivity of ceramic can be effectively reduced by introducing pores. It is worth mentioning that introducing small-sized pores is more conducive to heat insulation because they provide more interfaces for phonon scattering and inhibit convection heat transfer of gas molecules. For a given porosity, porous media with a higher proportion of large pores have a weaker reflection and scattering effect on heat transfer [54,55,56]. Even though the HEC-5 sample possesses higher porosity than the HEC-3 sample, the dual-porosity HECs, like the HEC-3 sample, have a thermal conductivity comparable to that of the single-pore HECs such as the HEC-5 sample. Thus, the dual-porosity structure improves the thermal insulation of porous HECs. Except for the high-entropy effect, the excellent compressive strength of the porous HECs is also ascribed to the dual-porosity structure. It is known that the pore sizes greatly influence stress concentrations. As the pore sizes grow larger, the stress concentration becomes more significant. The cracks under the action of the load are more likely to expand, which leads to lower strength [57,58]. For a given porosity, smaller pore sizes increase the number of pores, resulting in more crack deflecting for porous ceramics under load. This suggests that small-sized pores prolong the path of crack propagation and help load transfer, ultimately improving the compressive strength of the porous material [59].

## 4. Conclusions

The porous HECs have been fabricated by an in situ carbothermal reaction and pressureless sintering method. The pore structures were tuned by adding different amounts of SiO_2_ microspheres in the precursor powder.

(1) The prepared porous HECs exhibited a dual-porosity structure. The carbothermal reaction and sintering of ceramics precursor led to the formation of small pores of sizes 0.4–3 μm. The SiO_2_ microspheres resulted in the generation of large pores in the range of 20–50 μm. With the increase in the number of SiO_2_ microspheres from 0 wt.% to 20 wt.%, the small pores disappeared gradually, and the large pores appeared.
(2) The thermal conductivity of the porous HECs declined from 4.12 W·m^−1^ k^−1^ to 1.11 W·m^−1^ k^−1^ with the increase in porosity from 23.08% to 59.34%, while the compressive strength decreased from 133.1 MPa to 41.9 MPa. The obtained porous HECs showed competitive thermal insulation and excellent mechanical properties. The porous HECs are a promising family of materials for the TPS.
(3) The unique properties of the porous HECs are ascribed to the high-entropy effects and dual-porosity structures. The severe lattice distortions in the HECs lead to the low intrinsic thermal conductivity by reducing phonons’ mean free path and increasing phonon scattering. Moreover, this results in high compressive strength by inhibiting the crack generations and propagations. The dual-porosity structure possesses several small pores in high-porosity ceramics. These small pores are more efficient in scattering phonon thermal conductivity and inhibiting crack propagations, which are beneficial to improving the thermal insulation and mechanical properties of the porous HECs.

## Figures and Tables

**Table 1:** Porosity, density, and compressive strength of the obtained porous HECs.

Samples	Porosity (%)	Sintered Density (g·cm^−3^)	Compressive Strength (MPa)
HEC-1	23.08	7.29	133.1
HEC-2	28.67	6.74	121.5
HEC-3	36.92	5.76	103.2
HEC-4	47.23	4.72	82.7
HEC-5	59.34	3.97	41.9

**Table 2:** Thermal diffusivity (*α*), specific heat capacity (*C_p_*), and thermal conductivity (*k*) values for the porous HECs with different amounts of pore-forming agent.

Samples	*α* (mm^2^ S^−1^)	*C_p_* (J·g^−1^ k^−1^)	*k* (W·m^−1^ k^−1^)
HEC-1	2.68	0.21	4.12
HEC-2	1.94	0.22	2.96
HEC-3	1.36	0.25	1.97
HEC-4	1.07	0.28	1.42
HEC-5	0.92	0.30	1.11

## Data Availability

Not applicable.
